# Association between urinary organophosphate ester metabolite exposure and thyroid disease risk among US adults: National Health and Nutrition Examination Survey 2011-2014

**DOI:** 10.3389/fendo.2024.1329247

**Published:** 2024-02-09

**Authors:** Yuxin Lin, Ruipeng Lin, Weikang Wang, Manling Xie, Yun Li, Qian Zhang

**Affiliations:** ^1^ Department of Epidemiology and Health Statistics, School of Public Health, Fujian Medical University, Fuzhou, Fujian, China; ^2^ Laboratory Center, The Major Subject of Environment and Health of Fujian Key Universities, School of Public Health, Fujian Medical University, Fuzhou, China; ^3^ Food and Chemical Institute, Anhui Province Institute of Product Quality Supervision & Inspection, Hefei, China

**Keywords:** organophosphate esters, thyroid diseases, Weighted Quantile Sum (WQS) regression, Bayesian Kernel Machine Regression (BKMR), nutrition surveys, mixed exposure

## Abstract

**Background:**

Organophosphate esters (OPEs) may interfere with thyroid function, but the relationship between OPEs and thyroid disease remains unclear. This study aims to elucidate the relationship between OPEs exposure and thyroid disease risk in the general population in the United States.

**Method:**

Data were obtained from the 2011-2014 National Health and Nutrition Examination Survey cycle. All participants were tested for seven OPE metabolites in their urine and answered questions about whether they had thyroid disease through questionnaires. Logistic regression was employed to analyze the association between exposure to individual OPE metabolites and thyroid disease. Weighted Quantile Sum (WQS) regression modeling was utilized to assess exposure to mixed OPE metabolites and risk of thyroid disease. Bayesian kernel machine regression(BKMR) models to analyze the overall mixed effect of OPE metabolites.

**Result:**

A total of 2,449 participants were included in the study, 228 of whom had a history of thyroid disease. Bis(1,3-dichloro-2-propyl) phos (BDCPP), Diphenyl phosphate (DPHP) and Bis(2-chloroethyl) phosphate (BCEP) were the top three metabolites with the highest detection rates of 91.75%, 90.77% and 86.57%, respectively. In multivariate logistic regression models, after adjustment for confounding variables, individuals with the highest tertile level of BCEP were significantly and positively associated with increased risk of thyroid disease (OR=1.57, 95% CI=1.04-2.36), using the lowest tertile level as reference. In the positive WQS regression model, after correcting for confounding variables, mixed exposure to OPE metabolites was significantly positively associated with increased risk of thyroid disease (OR=1.03, 95% CI=1.01-1.06), with BCEP and DPHP having high weights. In the BKMR model, the overall effect of mixed exposure to OPE metabolites was not statistically significant, but univariate exposure response trends showed that the risk of thyroid disease decreased and then increased as BCEP exposure levels increased.

**Conclusion:**

The study revealed a significant association between exposure to OPE metabolites and an increased risk of thyroid disease, with BCEP emerging as the primary contributor. The risk of thyroid disease exhibits a J-shaped pattern, whereby the risk initially decreases and subsequently increases with rising levels of BCEP exposure. Additional studies are required to validate the association between OPEs and thyroid diseases.

## 1 Introduction

Organophosphate esters (OPEs) are a group of chemical flame retardants widely used in building materials, electronic products, furniture, textiles and many other products (1, 2). Since the new century, brominated flame retardants have been gradually banned and eliminated due to a series of reasons such as persistent environmental pollution and physiological toxicity, organophosphorus flame retardants as their main alternatives have been developed rapidly, and the global production and consumption of OPEs have been increasing (3–5). It is estimated that global production of OPEs will account for more than 20% of all flame retardants in the future (6). The bonding between OPEs materials is non-chemically bonded and is susceptible to release from the product into the environment through volatilization or wear during its production, transport application and recycling (6). Current studies have detected the presence of OPEs in environmental media such as air, water, dust, soil and sediment (7–10). Humans are inevitably exposed to OPEs pollution, which poses a potential health risk through inhalation, dietary intake and dermal contact (11–13). It has been reported that OPEs and their metabolites have been detected in human breast milk, serum and urine (14–16). After entering the body, most OPEs are easily metabolized into dialkyl or diaryl groups and various hydroxylation products. Therefore, urinary OPE metabolites are commonly used as biomarkers to quantify human OPE exposure (17, 18). Studies have found associations between OPEs metabolite exposure and multiple adverse health outcomes, such as cardiovascular disease (19), obesity (20), and chronic kidney disease (21). Because of their ubiquity and potential biotoxicity, it is important to reveal the adverse effects of OPEs exposure on broader health outcomes.

Thyroid disorders are common endocrine diseases with increasing incidence in recent years, including thyroid cancer and benign thyroid diseases (22). Thyroid cancer is the most commonly detected cancer in young people aged 15-29 years and is characterized by a significantly higher incidence in women than men, with 44,280 new cases of thyroid cancer expected to be diagnosed in 2021 (23, 24). Benign thyroid diseases include nodular goiter, hypothyroidism or hyperthyroidism. Hypothyroidism and hyperthyroidism are also common diseases that have adverse effects on the cardiovascular system, female reproductive function, and in severe cases can even be life-threatening (25). The prevention and control of thyroid diseases has always been a difficult problem, and its risk factors has not yet been clarified.

It has been observed in animal experiments that OPEs can interfere with thyroid function through various signaling pathways (26, 27), and the underlying toxicological mechanism may be related to competitive binding of thyroid hormone receptors and interference with thyroid hormone production (27, 28). Epidemiological studies have also found that OPEs can interfere with human thyroid function (29). Considering that common thyroid disorders are closely related to thyroid-related hormones, such as hyperthyroidism, hypothyroidism and thyroid cancer, the association between OPEs and thyroid disorders is beginning to attract attention (30). A case-control study found that OPEs exposure contributes to changes in thyroid function, which increases the risk of thyroid cancer (29). However, the association between OPEs and thyroid diseases remains unclear.

Furthermore, given that humans are often exposed to multiple OPEs at the same time, past studies have focused on the association between single pollutant exposures and specific health outcomes, and less concern has been given to the combined effects of various OPEs exposures on humans. With the development of statistical methods, two novel statistical strategies, Weighted Quantile Sum (WQS) regression and Bayesian Kernel Machine Regression (BKMR) models, have been applied to address this problem (31, 32). This study therefore explores the association between OPE metabolites exposure and thyroid disease risk in the general population, based on the National Health and Nutrition Examination Survey (NHANES).

## 2 Materials and methods

### 2.1 Study design and population

**Figure 1:**
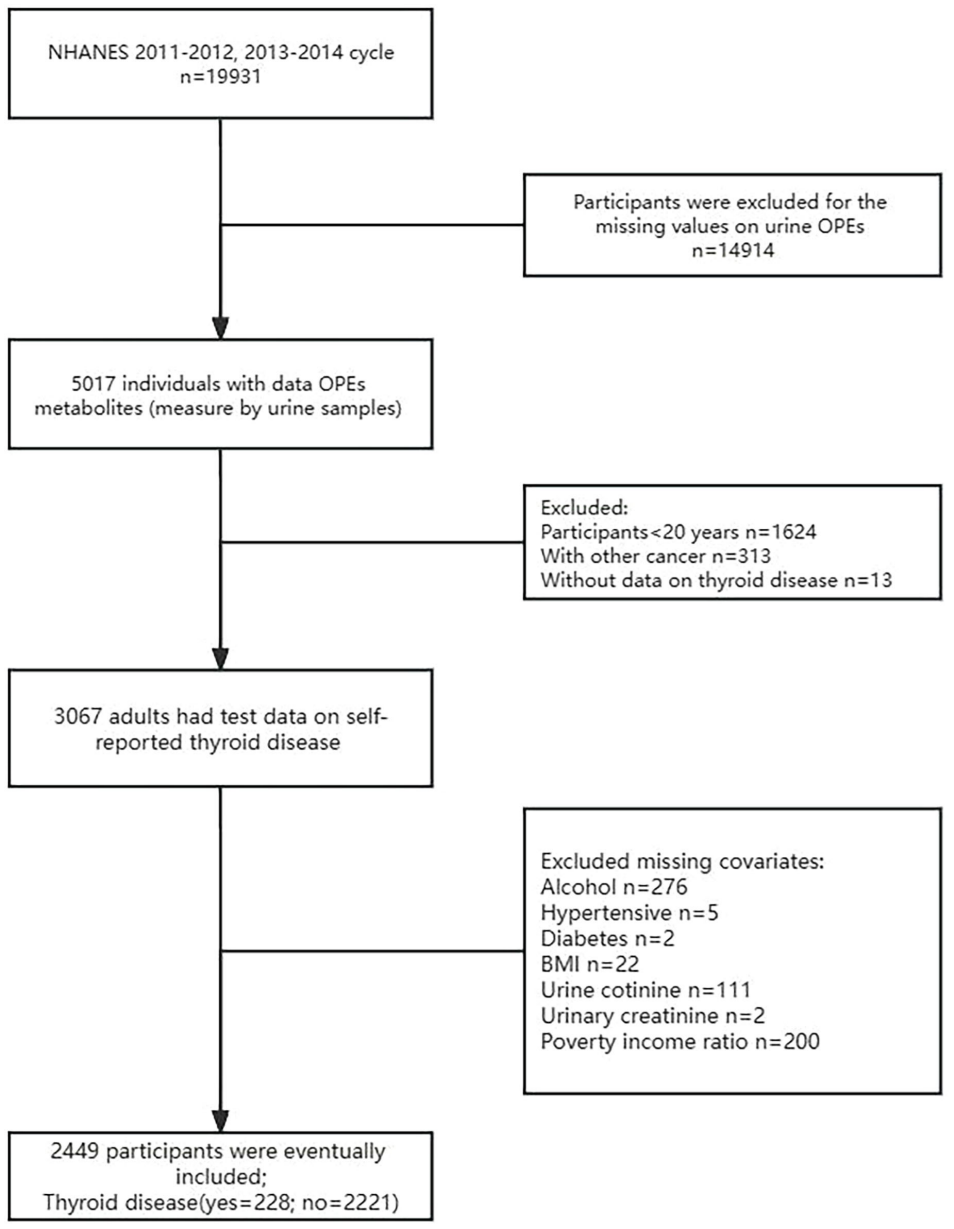
Flow chart of the participants’ selection.

NHANES is a cross-sectional national health nutrition assessment survey sponsored by the National Center for Health Statistics to investigate the nutritional and health status of the United States (US) population. The project uses a stratified multi-stage sampling design to recruit 5,000 American volunteers each year. Participants followed the voluntary principle of being interviewed, physically examined and biological samples collected. Survey data were published every two years on the official website starting in 1999. All procedures were approved by the National Center for Health Statistics Research Ethics Committee and written informed consent was obtained from the participants. We collected publicly accessible data from the NHANES project from 2011-2014 with the following inclusion criteria (1): Adults ≥ 20 years (2); Complete all urine OPE metabolites (3); Assessment of thyroid disease by means of a standardized questionnaire (4); Not suffering from any type of cancer other than thyroid cancer (5); No missing information on covariates. The detailed inclusion-exclusion process for participants is shown in **Figure 1**.

### 2.2 Measurements of urinary organophosphate ester metabolites

OPE metabolites were measured in urine using reversed phase high-performance liquid chromatography separation, and isotope dilution-electrospray ionization tandem mass spectrometry detection in the NHANES dataset. A total of seven OPE metabolites were detected in this study, namely: Diphenyl phosphate (DPHP); Bis(1,3-dichloro-2-propyl) phos (BDCPP); Bis(1-chloro-2-propyl) phosphate (BCPP); Bis(2-chloroethyl) phosphate (BCEP); Dibutyl phosphate (DBUP); Dibenzyl phosphate (DBZP); 2,3,4,5-tetrabromobenzoic acid (TBBA). The full names and abbreviations of the seven OPE metabolites tested in this study are shown in **Supplementary Table 1**. Their parent compounds are: Triphenyl phosphate (TPHP); Tris(1,3-dichloropropyl) phosphate (TDCPP); Tris(1-chloro-2-propyl) phosphate (TCPP); Tris(2-chloroethyl) phosphate (TCEP); Tributyl phosphate (TBP); Tribenzyl phosphate; 2-ethylhexyl-2,3,4,5- tetrabromobenzoate (TBB). Values below the limit of detection (LOD) are estimated by dividing the detection limit by the square root of 2 (LOD/√2). Metabolites with a detection rate of more than 50% were selected for subsequent statistical analysis. A detailed description of the experimental methods and procedures is available on the NHANES website (33).

### 2.3 Assessment of outcomes

Thyroid disease outcomes identified in self-reported “medical conditions” questionnaire in personal interview. The NHANES questionnaire item for thyroid disease is MCQ 160m: “Ever told you had thyroid problem”. If the answer was “yes”, the person was classified as a thyroid disease group; if the answer was “no”, the person was classified as a control group; if the answer was “don’t know” or refused to answer, the data were considered missing. In addition, patients with cancer types other than thyroid cancer were excluded according to items MCQ220 (Ever told you had cancer or malignancy) and MCQ230a (What kind of cancer). NHANES Interview guidelines, including specific interview questions, can be found in the **Supplementary Material**.

### 2.4 Covariates

Covariates were collected by NHANES through interviews or laboratory tests and included age, gender, race, marital status, body mass index (BMI), alcohol consumption status, history of hypertension, history of diabetes, cotinine level, urinary creatinine, education level, and family poverty income ratio (PIR). The NHANES questionnaire and interview guide used to assess the covariates are detailed in the **Supplementary Material**. Age, urinary creatinine and PIR were continuous variables. The race is classified into Mexican American, Other Hispanic, Non-Hispanic White, Non-Hispanic Black, Non-Hispanic Asian, Other Race. Marital status is classified as married or living with partner, widowed or divorced or separated, or never married. BMI is classified as underweight (<18.5 kg/m^2^), normal (18.5-24.9 kg/m^2^), overweight (25-29.9 kg/m^2^), obesity (≥30 kg/m^2^). Alcohol consumption status was referred as alcohol user and non-alcohol user (at least 12 alcoholic drinks per year or not). Serum cotinine level as a marker of nicotine exposure, classified as below the limit of detection (≤0.011 ng/mL), at or above the limit of detection (>0.011 ng/mL). According to the NHANES questionnaire, self-reported history of hypertension to determine hypertension and self-reported history of diabetes to determine diabetes. Education level is classified as less than high-school graduate, high-school graduate, college-level graduate.

### 2.5 Statistical analysis

Continuous variables were expressed as mean and standard deviation (SD) or median and inter-quartile range (IQR), and categorical variables were expressed as frequencies and percentages. The student’s t test (normal distribution) or Mann-Whitney test (skewed distribution) was used to compare thyroid disease cases and controls on continuous variables, and the chi-square test was used on categorical variables. Since OPE metabolites are non-normally distributed, they were log-transformed in this study for subsequent analysis.

Multivariate logistic regression models were used to assess the association between OPEs and thyroid disease risk. The groups were divided into high, medium and low exposure groups according to the tertile of urinary OPE metabolite concentrations. Odd ratios (ORs) and corresponding 95% confidence intervals (CIs) were calculated by comparing the medium and high OPEs exposure groups with the low exposure group. Model 1 adjusted for age, gender and race; Model 2 adjusted for age, gender, race, marital status, BMI, alcohol consumption status, history of hypertension, history of diabetes, cotinine level, and urinary creatinine. In addition, restricted cubic spline (RCS) curves were plotted to determine the potential non-linearity of the association between OPE metabolites and thyroid disease risk. Four nodes were set at the 5th, 35th, 65th and 95th percentiles in the model, and the reference value was set to the median of the ln-transformed OPE metabolites concentrations.

To assess the association between exposure to mixed OPEs and thyroid disease risk, we used WQS regression model (34). The model constructs a weighted index, namely the WQS index, to estimate the relationship between multiple exposures to environmental chemicals and outcomes. In addition, the model calculates the weight of the contribution of individual compounds to the overall effect of the mixture, thus identifying the important chemicals in the mixture. A detailed description of the method was presented in previous study (32). In this study, 40% of the data as the test set and the remaining 60% as the validation set.

Considering the potential interaction, non-linear effects and non-additive relationships between multiple environmental exposures, we used the BKMR model to assess the association between mixed OPEs exposure and thyroid disease risk. The method has been described in detail in previous study (31). In this study, Markov chain Monte Carlo algorithm was used to fit the BKMR model for 20,000 iterations. The BKMR model enables us to conduct the following analyses (1): calculate the effect of individual OPE metabolites on thyroid disease risk when the concentrations of other OPE metabolites were fixed at the median (2); compared the risk of thyroid disease for all metabolite concentrations of OPEs at a given percentile with the risk at the 50th percentile, and calculated the effect value of mixed exposure to OPEs (3); calculate the posterior inclusion probability (PIP), which indicates the relative importance of the effect of each OPE metabolite on the outcome, with a higher PIP indicating a greater contribution to the outcome (4); explore the pair-to-pair interaction of OPES metabolites when other metabolites are fixed at median levels. All statistical analyses were processed using R software (version 4.2.2). For RCS analysis, WQS analysis and BKMR analysis, the RMS package, the gWQS package and BKMR package were used, respectively. Two-sided *P < 0.05* was considered statistically significant.

## 3 Result

### 3.1 Characteristics of study participants

**Table 1** describes the demographic characteristics of the study subjects. A total of 2449 participants were included in the study, including 1230 (50.2%) men and 1219 (49.8%) women. The mean age of the enrolled subjects was 46.00 (32.00-61.00) years and the majority were non-Hispanic white or non-Hispanic black. 228 (9.3%) participants had self-reported thyroid problem, so they were classified as thyroid disease group, and the remaining subjects were control group. Participants in the thyroid disease group were younger and more likely to be female(*P<0.001*). There were also significant differences in race, BMI, cotinine level, urinary creatinine, Alcohol consumption status, marital status, history of hypertension and history of diabetes between the two groups (*P<0.05*).

**Table 1:** Characteristics of the study population (N=2449), NHANES, USA, 2011-2014.

Variable	Total	TD group	Control group	*P*
	N=2449	N=228	N=2221	
Age (years)	46.00 (32.00-61.00)	56.00 (45.25-69.00)	45.00 (31.00-60.00)	<0.001
Age group (years)				<0.001
20-	966 (39.4)	39 (17.1)	927 (41.7)	
40-	865 (35.3)	96 (42.1)	769 (34.6)	
60-	618 (25.2)	93 (40.8)	525 (23.6)	
Gender				<0.001
Male	1230 (50.2)	42 (18.4)	1188 (53.5)	
Female	1219 (49.8)	186 (81.6)	1033 (46.5)	
Race				0.001
Mexican American	280 (11.4)	21 (9.2)	259 (11.7)	
Other Hispanic	235 (9.6)	19 (8.3)	216 (9.7)	
Non-Hispanic White	1004 (41.0)	125 (54.8)	879 (39.6)	
Non-Hispanic Black	548 (22.4)	40 (17.5)	508 (22.9)	
Non-Hispanic Asian	305 (12.5)	16 (7.0)	289 (13.0)	
Other Race	77 (3.1)	7 (3.1)	70 (3.2)	
BMI (kg/m^2^)	27.9 (24.1-32.8)	29.80 (25.70-35.10)	27.70 (24.00-32.40)	0.001
BMI group (kg/m^2^)				0.002
<18.5	41 (1.7)	3 (1.3)	38 (1.7)	
18.5-	694 (28.3)	47 (20.6)	647 (29.1)	
25.0-	791 (32.3)	66 (28.9)	725 (32.6)	
30.0-	923 (37.7)	112 (49.1)	811 (36.5)	
PIR	2.02 (1.04-3.97)	1.96 (1.07-3.95)	2.02 (1.04-3.97)	0.97
Cotinine level				0.032
≥LOD	1760 (71.9)	150 (65.8)	1610 (72.5)	
<LOD	689 (28.1)	78 (34.2)	611 (27.5)	
Urinary creatinine	109.00 (61.00-166.00)	94.00 (47.25-149.75)	109.00 (62.00-167.00)	0.004
Alcohol consumption status				<0.001
≥12 drink/year	1808 (73.8)	146 (64.0)	1662 (74.8)	
<12 drink/year	641 (26.2)	82 (36.0)	559 (25.2)	
Education level				0.645
Less than high-school graduate	485 (19.8)	41 (18.0)	444 (20.0)	
High school graduate	540 (22.0)	48 (21.1)	492 (22.2)	
College level graduate	1424 (58.1)	139 (61.0)	1285 (57.9)	
Marital status				<0.001
Married or living with partner	1415 (57.8)	130 (57.0)	1285 (57.9)	
Widowed of divorced or separated	505 (20.6)	67 (29.4)	438 (19.7)	
Never married	529 (21.6)	31 (13.6)	498 (22.4)	
History of hypertension				<0.001
Yes	857 (35.0)	117 (51.3)	740 (33.3)	
No	1592 (65.0)	111 (48.7)	1481 (66.7)	
History of diabetes				0.002
Yes	283 (11.6)	40 (17.5)	243 (10.9)	
No	2110 (86.2)	179 (78.5)	1931 (86.9)	
Borderline	56 (2.3)	9 (3.9)	47 (2.1)	

Normally distributed continuous variables are expressed as mean and standard deviation, skewed continuous variables are expressed as median and interquartile range, and categorical variables are expressed as frequency and percentage. The student’s t test (normal distribution) or Mann-Whitney test (skewed distribution) was used to compare thyroid disease cases and controls on continuous variables, and the chi-square test was used on categorical variables. TD, thyroid diseases.

### 3.2 Measurements of urinary OPE metabolites

The distribution of urinary OPE metabolite concentrations is shown in **Table 2**. There were five OPE metabolites with detection rate >50%, which were BDCPP, DPHP, BCEP, DBUP and BCPP, with positive rates of 91.75%, 90.77%, 86.57%, 59.82% and 56.72%, respectively.

**Table 2:** Distribution of the OPE metabolite concentrations (N=2449).

OPE metabolites	LOD (µg/L)	Detection rate(%)	Mean (µg/L)	Percentile(µg/L)				
				5	25	50	75	95
DPHP	0.100	90.772	1.653	0.110	0.330	0.750	1.590	5.740
BDCPP	0.100	91.752	1.639	0.080	0.290	0.741	1.770	5.850
BCPP	0.100	56.717	0.359	0.070	0.071	0.130	0.310	1.100
BCEP	0.100	86.566	1.126	0.060	0.170	0.400	0.960	3.735
DBUP	0.100	59.820	0.221	0.040	0.071	0.120	0.300	0.570
DBZP	0.050	0.082	0.038	0.035	0.035	0.040	0.040	0.040
TBBA	0.050	4.777	0.045	0.035	0.035	0.040	0.040	0.040

### 3.3 Association between OPEs and thyroid disease risk by logistic regression analysis

**Table 3** shows the results of multivariate logistic regression analysis of the association between OPEs and thyroid disease risk. When OPE metabolites were used as continuous variables, increased concentrations of DPHP, BDCPP, BCPP, and DBUP were not significantly associated with thyroid disease risk, while the positive association between BCEP concentration and thyroid disease risk was marginally statistically significant. When OPE metabolites were used as categorical variables, no association was found between OPE metabolites and thyroid disease risk after adjusting for age, gender, and race (Model 1). However, after further adjustment for marital status, BMI, alcohol consumption status, history of hypertension, history of diabetes, cotinine level, and urinary creatinine, individuals with the highest tertile of BCEP exposure had a significantly increased risk of thyroid disease relative to the lowest tertile (OR=1.57, 95% CI=1.04-2.36). In addition, the trend test P value of BCEP was less than 0.05, suggesting that the risk of thyroid disease increased with BCEP levels.

**Table 3:** Association of OPE metabolites exposure and the risk of thyroid disease.

	Continuous	OR (95% CI)			*P _trend_^a^*
		Tertile 1	Tertile 2	Tertile 3	
Model 1					
DPHP	0.99(0.95-1.03)	Reference	1.31(0.89-1.92)	1.19(0.79-1.81)	0.657
BDCPP	1.00(0.96-1.04)	Reference	1.23(0.85-1.78)	0.85(0.54-1.33)	0.242
BCPP	1.10(0.98-1.23)	Reference	0.92(0.63-1.36)	0.92(0.63-1.35)	0.675
BCEP	1.02(0.99-1.05)	Reference	0.92(0.62-1.36)	1.56(1.05-2.33)	0.003
DBUP	1.06(0.73-1.52)	Reference	0.78(0.53-1.14)	0.81(0.56-1.15)	0.335
Model 2					
DPHP	0.98(0.94-1.03)	Reference	1.24(0.84-1.83)	1.08(0.69-1.68)	0.934
BDCPP	0.99(0.95-1.04)	Reference	1.14(0.78-1.66)	0.74(0.46-1.18)	0.088
BCPP	1.09(0.97-1.23)	Reference	0.89(0.60-1.32)	0.87(0.59-1.28)	0.491
BCEP	1.02(0.99-1.05)	Reference	0.95(0.64-1.40)	**1.57(1.04-2.36)**	**0.005**
DBUP	1.03(0.71-1.50)	Reference	0.75(0.51-1.10)	0.80(0.55-1.15)	0.296

Model 1: Adjusted for age, gender, race.

Model 2: Adjusted for age, gender, race, marital status, BMI, alcohol consumption status, history of hypertension, history of diabetes, cotinine level, and urinary creatinine.

a: The median value of each metabolite’s tertile was included in the regression model to test the trend.

Reference category: tertile 1.

Bold values indicate significant.

**Figure 2:**
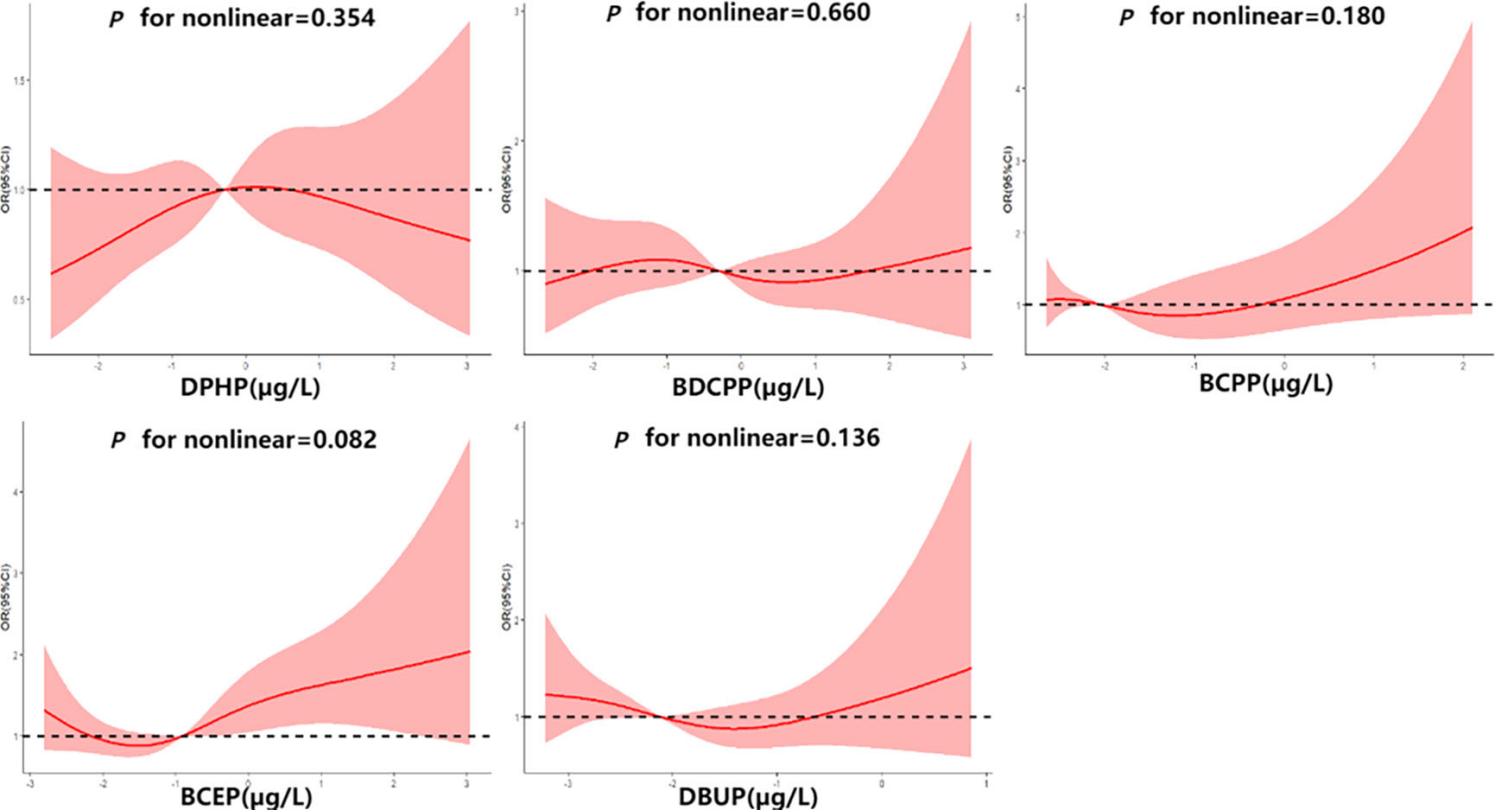
Association between ln-transformed urinary OPE metabolite concentrations and thyroid disease risk.

The RCS curves are shown in **Figure 2**. No non-linear relationship was observed between OPE metabolites and thyroid disease risk (*P* for nonlinear>0.05). In this study, the association between DPHP concentration and thyroid disease risk was observed as an inverted U-shaped curve, while BCEP was a J-shaped curve. When BCEP exposure increased to a certain concentration, the risk of thyroid disease increased, which was similar to the results of logistic regression model.

### 3.4 Association between OPEs and thyroid disease risk by WQS regression model

The WQS model analyzed the potential effect of mixed OPEs on thyroid disease. When analysis restricted in the positive direction, the WQS index was significantly positively associated with thyroid disease (OR=1.03, 95% CI=1.01-1.06, *P*=0.013). BCEP (65%) had the highest weight, followed by DPHP (35%), indicating that they play an important role in thyroid disease. When the analysis was restricted to the negative, there was no association found between the WQS index and thyroid disease (OR=1.00,95% CI=0.97-1.04, *P*=0.799).

### 3.5 Association between OPEs and thyroid disease risk by BKMR model

**Figure 3:**
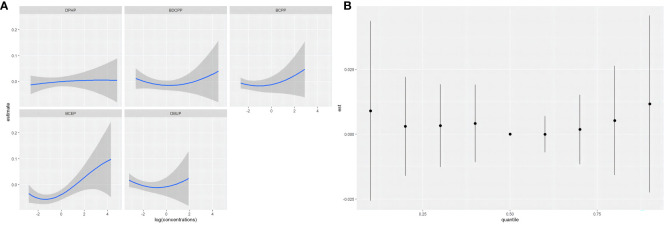
**(A)** Univariate exposure-response functions and 95% confidence intervals for each OPE metabolite, with the remaining OPE metabolites fixed at 50%; **(B)** Combined effect of BKMR model mixtures on thyroid disease when comparing all OPE metabolites at a specific percentile with all OPE metabolites at the 50th percentile; models were adjusted for age, gender, race, marital status, BMI, alcohol consumption status, history of hypertension, history of diabetes, cotinine level, and urinary creatinine.

**Figure 4:**
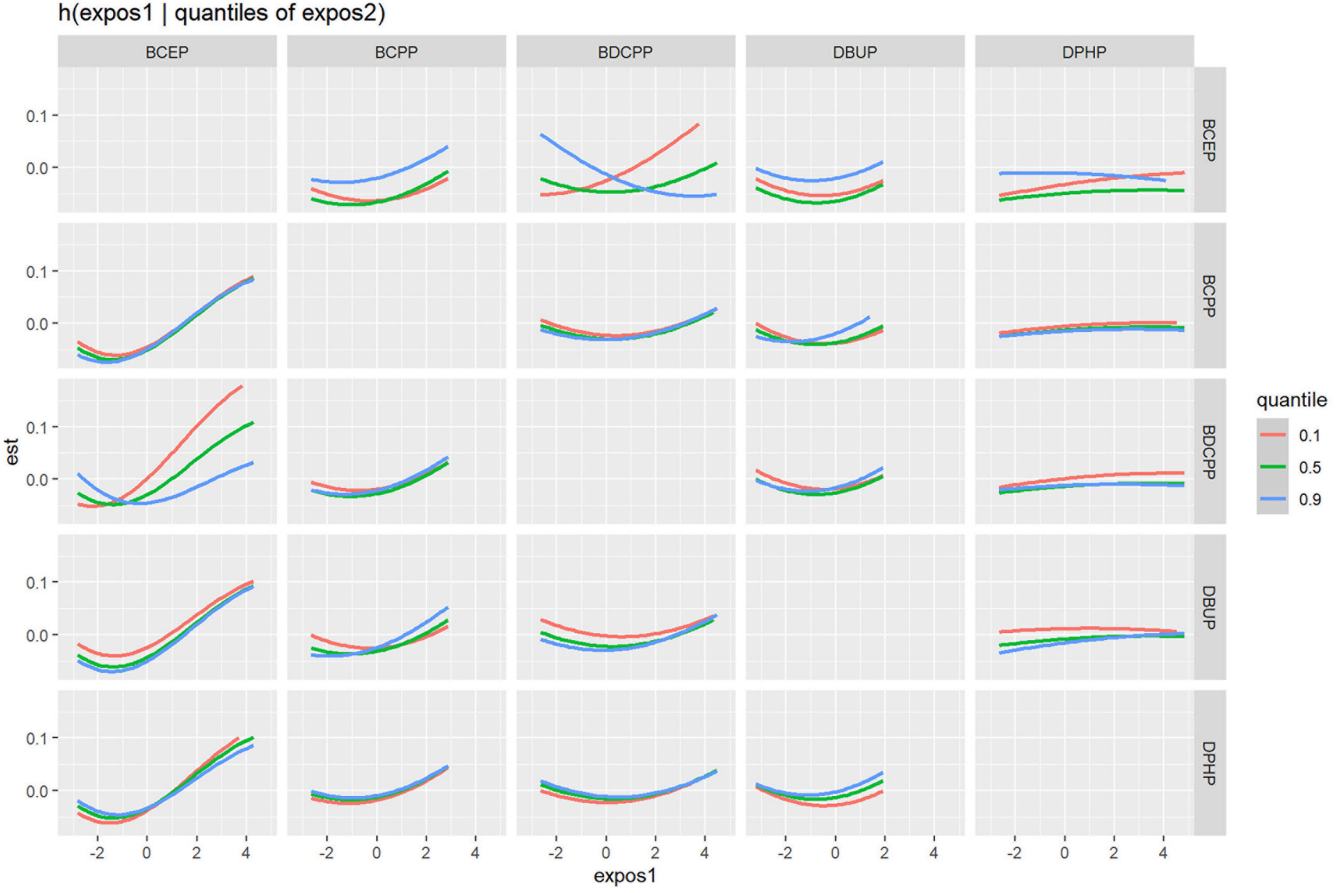
Association between exposure 1 with thyroid disease risk, while fixing exposure 2 at the 10th, 50th, and 90th quantiles (and fixing the remaining chemical exposure at the median level). Model was adjusted for age, gender, race, marital status, BMI, alcohol consumption status, history of hypertension, history of diabetes, cotinine level, and urinary creatinine.

When the other OPE metabolites were fixed at the median, the univariate exposure response trend for each OPE metabolite was shown in **Figure 3A**. The fit curve of BCEP exposure concentration and thyroid disease risk showed a “J” shape. As BCEP exposure levels increased, thyroid disease risk first decreased and then increased. The curves of the remaining OPE metabolites are flatter. The overall effect of OPEs is shown in **Figure 3B**, although not statistically significant, when all metabolites of OPEs were exposed at each percentile (except at the 60th percentile) and had an increased risk of thyroid disease compared with their exposure at the 50th percentile. And at the 70th percentile and above, there was a trend for thyroid disease to increase with increasing metabolites of OPEs. The highest PIP for thyroid disease was BCEP (PIP=0.52), followed by DBUP (PIP=0.36), and BCPP (PIP=0.32), indicating that BCEP plays an important contribution to thyroid disease risk. Then the bivariate expose-response interaction is also analyzed. In **Figure 4**, the columns represent one of the OPE metabolite exposures studied (“ exposure 1 “), each row represents “exposure 2” at the 10th, 50th, and 90th, while the other three metabolite exposures are fixed at the median concentration. It was observed that the fit curves of BCEP and thyroid disease risk clearly intersected with slope differences when BDCPP was at different levels (10th, 50th, and 90th percentiles), suggesting a potential interaction between BCEP and BDCPP exposure on thyroid disease risk.

## 4 Discussion

To the best of our knowledge, no previous studies have investigated the association between exposure to OPEs and the risk of thyroid disease. In this study, we investigated the relationship between urinary levels of OPEs and thyroid disease using multiple mixture analysis models. Overall, our findings were consistent across all analyzed models. The aggregated results demonstrated a positive association between urine OPE metabolites and the risk of thyroid disease. Notably, among the OPE mixtures, BCEP appeared to be the most significant contributor. Furthermore, our study also revealed significant interactions among different OPE mixtures, with a noteworthy negative interaction observed between BCEP and BDCPP in the BKMR model.

Generalized linear regression models, encompassing multivariate logistic regression and linear regression, are extensively employed in evaluating the health ramifications of chemical substances (35–37). Previous studies utilizing multivariate logistic regression and linear regression analyses have demonstrated that exposure to OPEs can induce changes in thyroid hormone levels (38, 39). These analyses often concentrate on a single chemical. However, multi-pollutant mixtures in the natural milieu can harbor intricate non-linear and non-cumulative associations with health (31). Disregarding the interactions between chemicals may result in spurious outcomes (35). The findings of multivariate logistic regression analysis revealed a favorable link between BCEP and susceptibility to thyroid disease. In this scenario, individual model studies yield less robust outcomes as the interactions between compounds is not considered. Moreover, chemical interaction cannot be discerned in a simplistic model.

Recently developed WQS regression and BKMR models, which better reflect the complexity of real-life multiple exposures. The WQS model can initially explore the link between overall compound mixture exposure and outcomes, and provide specific effect values and weighting of each compound’s contribution to outcomes that more closely resemble realistic human exposure to OPEs. The WQS model showed a significant positive association between urine OPE metabolites and thyroid disease, with the most contributing compound being BCEP, followed by DPHP. This result is consistent with the results of multivariate logistic regression analysis. The BKMR model can assess the overall hazard trends of mixed exposures of compounds at different exposure levels and can also represent the interactions between any two chemicals. In the BKMR model, it was observed that the risk of thyroid disease exhibited a J-shaped relationship with increasing levels of BCEP exposure. Additionally, there might exist an interaction between BCEP and BDCPP, influencing the risk of thyroid disease. When BDCPP was in the 10th percentile, the fitted curve of BCEP and the risk of thyroid disease exhibited a steeper slope compared to its presence in the 90th percentile. These findings partly explain the low weighting of BDCPP in the model.

The three models in our study provided similar results, with exposure to mixed OPEs having a tendency to increase the risk of thyroid disease and pointing to BCEP as the most significant compound responsible for this trend. Previous studies have established an association between OPEs exposure and thyroid hormone alterations, however few studies have clarified the association between OPEs and thyroid disease. Previous study found that tri-n-propyl phosphate, TCPP, TDCPP and TBEP in OPEs significantly increased the risk of thyroid cancer (29). Among them, TBEP is the parent compound of BCEP. This is similar to the results of our study.

The potential mechanisms by which OPEs interfere with thyroid function have not been fully elucidated, and evidence from animal and *in vitro* studies suggests that OPEs can induce thyroid dysfunction through a variety of signaling pathways. OPEs can affect certain gene expression and signaling pathways related to thyroid hormone (TH) synthesis, metabolism, transport and elimination in zebrafish embryos, leading to thyroid dysplasia (40). Binding of TH to thyroid hormone receptors is thought to be a major potential target for OPEs-induced thyroid destruction (41). It was found that OPEs can compete with TH to bind to membrane thyroid hormone receptors to enter cells, thereby inducing thyroid endocrine disruption. OPEs also competitively bind to zebrafish thyroxine transporter protein and thyroxine-binding globulin, thereby affecting the transport of TH in the blood, and it can interfere with thyroid peroxidase and thyroglobulin to inhibit TH synthesis (27).

The OPE metabolites that contributed most to thyroid disease in our study were BCEP. unfortunately, there are few studies on the effects of BCEP on thyroid function, and more research evidence is needed to explain the biological mechanisms leading to thyroid damage. Although the toxicity of BCEP has not been elucidated, the mechanism of toxicity of its parent compound, TCEP, may provide some clues. Animal studies have shown that TCEP exposure is associated with thyroid endocrine disruption and neurotoxicity (42, 43). TCEP has the potential to reduce plasma levels of TSH, T3 and thyroxine (T4) in freshwater fish and trigger oxidative stress in the organism (44). It was also found that TCEP caused significant changes in gene expression in the hypothalamic-pituitary-thyroid axis in the brain or liver of zebrafish (45). The same evidence was found in humans that OPE exposure was associated with altered thyroid function and an increased risk of thyroid cancer (29). A case-control study shows that TCEP exposure is associated with the development and severity of papillary thyroid cancer (46).

In addition, animal experiments have shown that exposure to OPEs can induce oxidative stress and lipid peroxidation of DNA damage (47). Reported urinary metabolites of OPEs (e.g., BCEP, DPHP, DBUP) positively correlate with the concentration of 8-hydroxy-2′-deoxyguanosine (8-OHdG), a marker of DNA oxidative stress, suggesting that human exposure to OPEs may lead to DNA oxidative stress (48). Thyroid hormones are important regulators of antioxidants, and oxidative stress is closely related to thyroid function (49). In the mother-infant population it was observed that oxidative stress may be involved in the association between maternal OPEs exposure and maternal and neonatal TSH alterations, with 8-OHdG being the main mediator of the positive association between neonatal TSH and DPHP (50). In this study DPHP was the second contributing compound in the WQS model, and DPHP causes oxidative stress damage may provide some evidence to explain that it causes thyroid function impairment.

Our study has several advantages. Previous studies have predominantly focused on the association between single pollutant exposure and specific health outcomes, while our study emphasizes the comprehensive impact of mixed exposure to OPEs on humans. Additionally, we employ a series of mixed analysis models, including multivariate logistic regression, WQS model and BKMR model to comprehensively evaluate the association between OPE mixtures and the risk of thyroid disease. Logistic regression is inadequate for evaluating the comprehensive effects of OPE mixtures since their effects cannot be simply calculated as the sum of individual effects (51). The WQS model can investigate the effects of mixed exposure burdens on outcomes in one direction at a time, but it relies on meeting directional uniformity assumptions and assumes linear and cumulative effects of individual exposures. The BKMR model is a valuable statistical tool for examining the effects of combined mixtures, offering linear or nonlinear response functions and visualizations to enhance the identification of key pollutants. However, inferring exposure-response functions by fixing other chemicals at a certain level cannot estimate the effects of common exposure patterns with varying chemical levels. Simultaneously using these models allows for consideration of their advantages and disadvantages in order to elucidate the interactions between chemical mixtures.

Nevertheless, this study has several limitations. Firstly, the study design employed a cross-sectional approach, which precluded the exploration of a causal relationship between OPE exposure and thyroid disease. Secondly, we used unweighted data in our analysis due to concerns of over-adjustment bias and suitability of sampling weight for complex statistical models. However, this decision may limit the generalizability of our findings. Thirdly, participants underwent only a single urine sample measurement, neglecting potential fluctuations in OPE metabolite levels over time. Moreover, fixed values were utilized to substitute for OPE metabolite levels below the detection limit, leading to an inaccurate portrayal of individual OPE exposure. Fourthly, the outcome relied on self-reported cases of thyroid disease, thereby introducing the potential for recall bias. Lastly, the study’s restricted analysis of only five OPE metabolites fails to capture the comprehensive spectrum of human exposure.

## 5 Conclusion

The study revealed an association between OPE metabolites exposure and increased risk of thyroid disease, with BCEP being the most significant substance. The risk of thyroid disease exhibited a J-shaped pattern, decreasing initially and then increasing with higher levels of BCEP exposure. Our study initially explored the effect of OPEs exposure on thyroid disease, and longitudinal and experimental studies with larger samples are needed to validate our preliminary results and elucidate the underlying mechanisms of OPEs exposure and thyroid disease.

## Data availability statement

Publicly available datasets were analyzed in this study. This data can be found here: https://www.cdc.gov/nchs/nhanes/index.htm.

## Ethics statement

The study protocols were approved by the institutional review board of the National Center for Health Statistics. The studies were conducted in accordance with the local legislation and institutional requirements. The data are available from the US National Health and Nutrition Examination Survey (NHANES) 2011–2014. Written informed consent for participation was not required from the participants or the participants’ legal guardians/next of kin in accordance with the national legislation and institutional requirements.

## Author contributions

YXL: Conceptualization, Data curation, Formal analysis, Methodology, Software, Validation, Visualization, Writing – original draft, Writing – review & editing. RL: Conceptualization, Data curation, Formal analysis, Methodology, Software, Validation, Visualization, Writing – original draft, Writing – review & editing. WW: Formal analysis, Methodology, Software, Visualization, Writing – original draft, Writing – review & editing. MX: Software, Visualization, Writing – review & editing. YL: Validation, Writing – review & editing. QZ: Conceptualization, Funding acquisition, Supervision, Validation, Writing – review & editing.
