# Supplementary material for: Association between urinary organophosphate ester metabolite exposure and thyroid disease risk among US adults: National Health and Nutrition Examination Survey 2011-2014
Source: Front Endocrinol (Lausanne). 2024 Feb 9;15:1329247. doi: 10.3389/fendo.2024.1329247 (PMC10884265; doi:10.3389/fendo.2024.1329247)
Supplement: Supplementary file 1 [file Table_1.docx]

Supplementary Material

**Table S1 Abbreviations and full names of OPE types detected in this study**

| **Full name** | **Abbreviation** |
| --- | --- |
| Diphenyl phosphate | DPHP |
| Bis(1,3-dichloro-2-propyl) phos | BDCPP |
| Bis(1-chloro-2-propyl) phosphate | BCPP |
| Bis(2-chloroethyl) phosphate | BCEP |
| Dibutyl phosphate | DBUP |
| Dibenzyl phosphate | DBZP |
| 2,3,4,5-tetrabromobenzoic acid | TBBA |

**NHANES Interview Questionnaire and Interview Guide**

The interview questionnaire and interview guide used in this study to assess thyroid disease as well as covariates were obtained from the US National Health and Nutrition Examination Survey (https://www.cdc.gov/nchs/nhanes/index.htm)

**Interview Setting and Mode of Administration**

The family and sample person demographics questionnaires were asked, in the home, by trained interviewers using Computer-Assisted Personal Interviewing (CAPI) system. The respondent selected the language of interview (English or Spanish) or requested that an interpreter be used. Hand cards showing response choices or information that survey participants needed to answer the questions were used for some questions. The hand cards were printed in English, Spanish, Mandarin Chinese (both traditional and simplified), Korean, and Vietnamese. The interviewer directed the respondent to the appropriate hand card during the interview. When necessary, the interviewer further assisted the respondent by reading the response choices listed on the hand cards.

**Quality Assurance & Quality Control**

Persons 16 years and older and emancipated minors were interviewed directly. A proxy provided information for survey participants who were under 16 and for participants who could not answer the questions themselves.

The CAPI system is programmed with built-in consistency checks to reduce data entry errors. CAPI also uses online help screens to assist interviewers in defining key terms used in the questionnaire.

After collection, interview data were reviewed by the NHANES field office staff for accuracy and completeness of selected items. The interviewers were required to record interviews periodically and the recorded interviews were reviewed by NCHS staff and interviewer supervisors.

**Interview Questions**

**Variable Name:** MCQ160m

**SAS Label:** Ever told you had thyroid problem

**English Text:** Has a doctor or other health professional ever told {you/SP} that {you/s/he} . . .had another thyroid (thigh-roid) problem?

**Target:**Both males and females 20 years - 150 years

| Code or Value | Value Description |
| --- | --- |
| 1 | Yes |
| 2 | No |
| 7 | Refused |
| 9 | Don't know |
| . | Missing |

**Variable Name:** MCQ220

**SAS Label:** Ever told you had cancer or malignancy

**English Text:** {Have you/Has SP} ever been told by a doctor or other health professional that {you/s/he} had cancer or a malignancy (ma-lig-nan-see) of any kind?

**Target:** Both males and females 20 years - 150 years

| Code or Value | Value Description |
| --- | --- |
| 1 | Yes |
| 2 | No |
| 7 | Refused |
| 9 | Don't know |
| . | Missing |

**Variable Name:** MCQ230a

**SAS Label:** What kind of cancer

**English Text:** What kind of cancer was it?

**English Instructions:** Enter up to 3 kinds. If respondent offers more than 3, enter 66 as the 4th response. Capi instructions: Allow up to 3 entries. Allow 'more than 3 kinds' (code 66) only as 4th entry.

**Target:** Both males and females 20 years - 150 years

| Code or Value | Value Description | Code or Value | Value Description |
| --- | --- | --- | --- |
| 10 | Bladder | 27 | Nervous system |
| 11 | Blood | 28 | Ovary (ovarian) |
| 12 | Bone | 29 | Pancreas (pancreatic) |
| 13 | Brain | 30 | Prostate |
| 14 | Breast | 31 | Rectum (rectal) |
| 15 | Cervix (cervical) | 32 | Skin (non-melanoma) |
| 16 | Colon | 33 | Skin (don't know what kind) |
| 17 | Esophagus (esophageal) | 34 | Soft tissue (muscle or fat) |
| 18 | Gallbladder | 35 | Stomach |
| 19 | Kidney | 36 | Testis (testicular) |
| 20 | Larynx/ windpipe | 37 | Thyroid |
| 21 | Leukemia | 38 | Uterus (uterine) |
| 22 | Liver | 39 | Other |
| 23 | Lung | 66 | More than 3 kinds |
| 24 | Lymphoma/ Hodgkin's disease | 77 | Refused |
| 25 | Melanoma | 99 | Don't know |
| 26 | Mouth/tongue/lip | . | Missing |

**Covariates：**Age

**Variable Name:** RIDAGEYR

**SAS Label:** Age in years at screening

**English Text:** Age in years of the participant at the time of screening. Individuals 80 and over are topcoded at 80 years of age.

**Target:** Both males and females 0 years - 150 years

| Code or Value | Value Description |
| --- | --- |
| 0 to 79 | Range of Values |
| 80 | 80 years of age and over |
| . | Missing |

**Covariates：**Gender

**Variable Name:** RIAGENDR

**SAS Label:** Gender

**English Text:** Gender of the participant.

**Target:** Both males and females 0 years - 150 years

| Code or Value | Value Description |
| --- | --- |
| 1 | Male |
| 2 | Female |
| . | Missing |

**Covariates:** Race

**Variable Name:** RIDRETH3

**SAS Label:** Race/Hispanic origin w/ NH Asian

**English Text:** Recode of reported race and Hispanic origin information, with Non-Hispanic Asian Category

**Target:** Both males and females 0 years - 150 years

| Code or Value | Value Description |
| --- | --- |
| 1 | Mexican American |
| 2 | Other Hispanic |
| 3 | Non-Hispanic White |
| 4 | Non-Hispanic Black |
| 6 | Non-Hispanic Asian |
| 7 | Other Race - Including Multi-Racial |
| . | Missing |

**Covariates：**Marital status

**Variable Name:** DMDMARTL

**SAS Label:** Marital status

**English Text:** Marital status

**Target:** Both males and females 20 years - 150 years

| Code or Value | Value Description |
| --- | --- |
| 1 | Married |
| 2 | Widowed |
| 3 | Divorced |
| 4 | Separated |
| 5 | Never married |
| 6 | Living with partner |
| 77 | Refused |
| 99 | Don't Know |
| . | Missing |

**Covariates :**Alcohol consumption status

**Variable Name:** ALQ101

**SAS Label:** Had at least 12 alcohol drinks/1 year?

**English Text:** The next questions are about drinking alcoholic beverages. Included are liquor (such as whiskey or gin), beer, wine, wine coolers, and any other type of alcoholic beverage. In any one year, {have you/has SP} had at least 12 drinks of any type of alcoholic beverage? By a drink, I mean a 12 oz. beer, a 5 oz. glass of wine, or one and half ounces of liquor.

**Target:** Both males and females 18 years - 150 years

| Code or Value | Value Description |
| --- | --- |
| 1 | Yes |
| 2 | No |
| 7 | Refused |
| 9 | Don't know |
| . | Missing |

**Covariates:** History of hypertension

**Variable Name:** BPQ020

**SAS Label:** Ever told you had high blood pressure

**English Text:** {Have you/Has SP} ever been told by a doctor or other health professional that {you/s/he} had hypertension, also called high blood pressure?

**Target:** Both males and females 16 years - 150 years

| Code or Value | Value Description |
| --- | --- |
| 1 | Yes |
| 2 | No |
| 7 | Refused |
| 9 | Don't know |
| . | Missing |

**Covariates:** History of diabetes

**Variable Name:** DIQ010

**SAS Label:** Doctor told you have diabetes

**English Text:** The next questions are about specific medical conditions. {Other than during pregnancy, {have you/has SP}/{Have you/Has SP}} ever been told by a doctor or health professional that {you have/{he/she/SP} has} diabetes or sugar diabetes?

**English Instructions:** Capi instruction: If sp age < 15, display "Have sp" For the first display and "Sp has" For the second display. If sp is female and age >= 20, display "Other than during pregnancy, {have you/has sp}".

**Target:** Both males and females 1 years - 150 years

| Code or Value | Value Description |
| --- | --- |
| 1 | Yes |
| 2 | No |
| 3 | Borderline |
| 7 | Refused |
| 9 | Don't know |
| . | Missing |

**Covariates:** Education level

**Variable Name:** DMDEDUC2

**SAS Label:** Education level - Adults 20+

**English Text:** What is the highest grade or level of school {you have/SP has} completed or the highest degree {you have/s/he has} received?

**English Instructions:** Hand card dmq1 read hand card categories if necessary enter highest level of school

**Target:** Both males and females 20 years - 150 years

| Code or Value | Value Description |
| --- | --- |
| 1 | Less than 9th grade |
| 2 | 9-11th grade (Includes 12th grade with no diploma) |
| 3 | High school graduate/GED or equivalent |
| 4 | Some college or AA degree |
| 5 | College graduate or above |
| 7 | Refused |
| 9 | Don't Know |
| . | Missing |

**Covariates:** Family poverty income ratio

**Variable Name:** INDFMPIR

**SAS Label:** Ratio of family income to poverty

**English Text:** A ratio of family income to poverty guidelines.

**Target:** Both males and **females** 0 years - 150 years

| Code or Value | Value Description |
| --- | --- |
| 0 to 4.99 | Range of Values |
| 5 | Value greater than or equal to 5.00 |
| . | Missing |
